# Plasma neurofilament light chain protein as a predictor of days in delirium and deep sedation, mortality and length of stay in critically ill patients

**DOI:** 10.1016/j.ebiom.2022.104043

**Published:** 2022-05-06

**Authors:** Valerie J Page, Leiv Otto Watne, Amanda Heslegrave, Allan Clark, Daniel F McAuley, Robert D Sanders, Henrik Zetterberg

**Affiliations:** a Department of Anaesthesia, Intensive Care Unit, Watford General Hospital, Vicarage Road, Watford WD18 0HB, UK; b Faculty of Medicine, Imperial College, South Kensington Campus, London SW7 2AZ, UK; c Department of Geriatric Medicine, Oslo Delirium Research Group, Oslo University Hospital, Oslo, Norway; d Department of Neurodegenerative Disease, UCL Queen Square Institute of Neurology, London, UK; e UK Dementia Research Institute at UCL, London, UK; f Norwich Clinical Trials Unit, University of East Anglia, Norwich, UK; g Wellcome-Wolfson Institute for Experimental Medicine, The Queen's University of Belfast, Health Sciences Building, Belfast, Northern Ireland, UK; h Royal Victoria Hospital, Belfast Health and Social Care Trust, Grosvenor Road, Belfast, Northern Ireland, UK; i Sydney Medical School/Central Clinical School, The University of Sydney, NSW 2006, Australia; j Department of Psychiatry and Neurochemistry, Institute of Neuroscience and Physiology, the Sahlgrenska Academy at the University of Gothenburg, Mölndal, Sweden; k Clinical Neurochemistry Laboratory, Sahlgrenska University Hospital, Mölndal, Sweden; l Hong Kong Center for Neurodegenerative Diseases, Hong Kong, China

**Keywords:** Neurofilament light protein, Delirium, Intensive care, Critical care, Biomarker

## Abstract

**Background:**

Delirium predicts poor outcomes, however identifying patients with the worst outcomes is challenging. Plasma neurofilament light protein (NfL) is a sensitive indicator of neuronal damage. We undertook an exploratory observational study to determine the association between plasma NfL and delirium in the critically ill.

**Methods:**

MoDUS was a randomised placebo-controlled delirium trial of simvastatin done in an UK adult general ICU. We measured NfL levels in plasma samples using a Single molecule array (Simoa) platform. We explored associations between patient's plasma NfL levels and number of delirium days, and clinical outcomes. The control group for baseline NfL were preoperative patients undergoing major surgery.

**Findings:**

The majority of critically ill patients already had a high NfL level on admission. Patients with higher plasma NfL levels at days one and three spent more days in delirium or deep sedation. Patients with zero or one day in delirium or deep sedation had day one mean concentrations of 37.8 pg/ml (SD 32.6) compared with 96.5 pg/ml (SD 106.1)) for patients with two days or more, p-value 0.002 linear mixed effects model.

Survivors discharged before 14 days had lower mean plasma NfL concentrations compared to those with longer hospital stays and/or who died within six months. The area under ROC curve for predicting death within six months using day one NfL was 0.81 (0.7,0.9).

**Interpretation:**

Measurement of plasma NfL within three days of admission may be useful to identify those patients with worse clinical outcomes, and as an enrichment strategy for future delirium interventional trials in the critically ill.

**Funding:**

Alzheimer's Society UK, UK Dementia Research Institute.

**Research in context**

**Evidence before the study**

We searched PubMed, MEDLINE, and the Cochrane Database of Systematic Reviews without language or date restrictions for published research that assessed the use of blood levels of neurofilament light protein as a delirium biomarker for mechanically ventilated patients in intensive care units. The most recent search was done on 14th September 2021. We searched PubMed with the key words “intensive care”, “critical care”, “neurofilament light protein”. We included only studies published in English in adult patients.

There were no studies in critically ill patients exploring the association between plasma or serum levels of neurofilament light protein and delirium.

Studies in blood levels of neurofilament light protein and outcomes have been published in different critically ill patient populations including post cardiac arrest patients, stroke patients, patients with COVID-19 and patients with sepsis. They demonstrated blood levels of neurofilament light protein are related to patient outcomes including mortality and neurological outcomes. A cohort study in elective postoperative patient population undergoing major surgery showed neurofilament light protein levels rose more sharply in patients who developed delirium, and levels were proportionate to delirium severity. A case control study in a similar population showed patients who developed delirium on the second postoperative day had higher preoperative plasma levels of neurofilament light protein.

**Added value of the study**

This study showed that neurofilament light protein levels on admission were elevated in over 82% of critically ill patients demonstrating acute neuronal damage, and were reflected in the number of delirium and deep sedation days. Levels were significantly elevated in sicker patients and those admitted with sepsis as compared to patients admitted without sepsis. The levels continued to rise in those patients who were still in the ICU up to 28 days.

**Implications of all the available evidence**

Critically ill patients with higher plasma neurofilament light protein levels are more likely to develop delirium or be deeply sedated for more than two days. By using neurofilament light protein levels in delirium research there is the potential to enrich the study population with patients more likely to respond to an intervention. A plasma neurofilament light concentration taken early in a patients critical care admission would also help identify patients who are likely to have a prolonged hospital stay or die within six months.

More research is needed to understand the time course of neurofilament levels and how they reflect ongoing damage, and relate to the start and progression of recovery.

Alt-text: Unlabelled box

## Introduction

Delirium is a clinical syndrome characterised by acute brain dysfunction and associated with worse outcomes including long-term cognitive impairment, increased length of hospital stay and costs.^1^, ^2^, ^3^ Critical care delirium is associated with new onset dementia, however it is not known if delirium pathology results in neuronal damage as opposed to the precipitating causes.^4^ Biomarkers have a role in helping unravel pathophysiological pathways, with potential as diagnostic and/or prognostic disease markers.

Studies on biomarkers of brain cell injury have conveyed conflicting results in terms of their association with delirium. Neurofilament light (NfL) is a neuronal scaffolding protein, which is released into the extracellular space upon neuroaxonal damage.^5^ Levels can be accurately measured in blood samples using ultra-sensitive Single molecule array (Simoa) technology. Plasma or serum can be used for quantification of blood NfL and NfL levels between them are highly correlated. Serum NfL levels increase during aging and are elevated in various neurologic conditions, including traumatic brain injury, multiple sclerosis, and neurodegenerative diseases.^6^, ^7^, ^8^, ^9^, ^10^, ^11^ In cognitively normal older adults undergoing elective surgery, peak delirium severity on the second post-operative day was associated with higher Nfl concentrations in both serum and CSF compared with patients who had no delirium.^12^ The correlation between serum and CSF NfL supports the use of NfL as a systemic biomarker in delirium studies.^13^

A study in 114 patients recruited to a prospective biomarker study in patients undergoing major elective surgery showed plasma NfL rose more sharply in patients with delirium postoperatively than patients without, and the NfL level rose proportionately to delirium severity.^12^ Similarly in a case-control study of patients undergoing major surgery showed those who developed delirium post-operatively on day two had higher levels of NfL pre-operatively.^14^ The NfL levels in those patients who developed delirium were still elevated at one month after hospitalization.

With regard to long-term outcomes in critically ill patients serum NfL level taken at 24 h after cardiac arrest is a highly predictive marker of long-term poor neurologic outcome. It has significantly greater performance than other biochemical serum markers, with greater sensitivity for poor outcome compared with routine electroencephalogram, somatosensory-evoked potentials, brain computed tomography, and brainstem reflexes.^15^ In addition elevated blood NfL in critically ill patients with COVID-19 is associated with longer hospital stays, and worse functional outcomes.^16^ Similarly outside of critical care patients with multiple sclerosis and a high baseline blood level of NfL suffer a more rapid progression of their disease than patients with lower NfL blood levels at the time of diagnosis.^7^ In traumatic brain injury patients blood NfL levels have been shown to correlate with functional outcomes and MRI atrophy.^9^

We undertook an exploratory observational study primarily to determine the association between plasma NfL and days spent in delirium or deep sedation in the critically ill using sequential samples collected during a randomised trail of simvastatin to modify delirium (the MoDUS study). Our hypotheses are based on the understanding that neurofilament light protein (NfL) is a marker of neuronal injury. They are that critically ill patients who spend more days in delirium with or without days in deep sedation will have higher levels of plasma NfL during ICU admission, that NfL levels will change throughout ICU admission and higher levels of NfL will be associated with worse clinical outcomes.

## Methods

### Ethics

MoDUS was approved by a national research ethics committee (12/NE/0383) and registered on the International Standard Randomised Controlled Trial Registry (ISRCTN89079989). Patients or their representatives written informed consent included the storage of samples for use in future research approved by an ethics committee, and was obtained for this sample analysis study (20/WA/0204).

### Study design and participants of MoDUS

We present a basic analysis of sequential neurofilament light protein levels (NfL) in the plasma of critically ill patients who participated in the previously reported MoDUS trial. MoDUS was a double-blind, placebo-controlled randomised delirium interventional trial conducted in a general adult intensive care unit (ICU) at Watford General Hospital.^17^ Critically ill adult patients were eligible if they needed mechanical ventilation within 72 h of admission. Patients were randomly assigned (1:1) to receive either simvastatin 80mgs or placebo daily for up to a maximum of 28 days treatment.

### Control group participants

Our control group for baseline NfL levels (day 1) were recruited to two prospective perioperative cohort studies registered with ClinicalTrials.gov (NCT03124303 ongoing, NCT02926417 completed) and approved by the University of Wisconsin-Madison Institutional Review Board (2015-0374, 2015-0960). The dataset included all participants with complete data who did not develop post-operative delirium.

### Procedures in MoDUS

Patients were enrolled within 72 h of needing mechanical ventilation and received once daily enteral simvastatin 80mgs or identical placebo tablets. The first dose was given as soon as possible following randomization and subsequent doses were administered each morning beginning the following day. Study drug was continued until day 28, discharge from ICU, death, or development of a contraindication to continued statin therapy.

Participant baseline demographic characteristics were recorded at the time of enrolment including APACHE II (Acute Physiology and Chronic Health Evaluation) score to assess severity of illness, the PRE-DELIRIC (PREdiction of DELIRium in ICu patients) delirium prediction model score to assess predicted risk of delirium and the Informant Questionnaire on Cognitive Decline in the Elderly (IQCODE) a surrogate assessment of cognitive impairment.^18^,^19^ Routine data regarding level of arousal, delirium screening and organ support were recorded daily while the patient remained in ICU up to a maximum of 28 days.

### Outcomes of MoDUS

The original primary outcome of MoDUS was number of days alive without delirium and without coma (deep sedation) at day 14. Patients were defined as delirious if they were assessed with a RASS sedation score of –2 to +4, where RASS -2 equals opening eyes to voice up to +4 as combative, and screened positive for delirium by the bedside nurse using the confusion assessment method-ICU (CAM-ICU).^20^ Patients with a lower RASS score of –3 to –5 were classified as if in a coma, irrespective of whether the state was induced by deep sedation, and therefore unable to be assessed (UTA) for delirium.^21^

Secondary outcomes included mortality, ventilator-free days, length of ICU and hospital stay, and cognitive function. Cognitive outcomes at six months were assessed by use of the Brief Test of Adult Cognition by Telephone (BTACT). The BTACT assesses multiple dimensions central for effective cognitive functioning including episodic memory, working memory, reasoning, verbal fluency, and executive function.^22^ There were no differences in any outcomes by treatment allocation as assessed in the original publication.

### Procedures

Plasma samples were taken at baseline prior to study drug administration (day 1) and on day 3, 7, 14 and 28 from all patients as long as they were inpatients on the ICU. Samples were not taken from study patients once they were discharged to the ward. They were processed, divided into aliquots, and frozen and stored at -20 °C before being transported frozen, on dry ice, to Queens University Belfast to be stored at – 80 °C according to standardized procedures. One 0.5 ml aliquot of plasma from each specimen was transported, frozen, on dry ice, to the UK Dementia Research Institute Fluid Biomarker Laboratory at UCL where it was thawed immediately prior to analysis. All MoDUS samples were analysed in the UK Dementia Research Institute Fluid Biomarker Laboratory using the same method and one batch of reagents.

### Assay procedures

Plasma NfL concentration was measured with the commercially available NF-Light assay on an ultrasensitive Single molecule array (Simoa) platform, as described by the manufacturer (Quanterix Corp, Billerica, MA). All samples were measured in duplicate. In addition, quality control samples are measured in duplicate on each of the runs used to complete the study. Intra- and inter-assay coefficients of variation are below 10% for this assay.

### Procedures control group study

Adult patients were recruited who were scheduled for major elective non-intracranial, non-cardiac surgery. Plasma samples were collected preoperatively and stored at – 80 °C. They were sent to the University of Gothenburg for analysis of NfL, using the same Simoa method (Quanterix, Billerica, MA), and similar assay performance (CVs <10%) as used in the MoDUS sample analysis undertaken at the UK Dementia Research Institute Fluid Biomarker Laboratory at University College London.

These results have been reported before and are included purely to give a crude estimation on how the MoDUS group relates to a control group representative of hospital patients without acute illness in terms of blood NfL concentration.^13^ Postoperatively participants underwent delirium assessments with the Confusion Assessment Method (CAM)/ 3D-CAM, or the CAM-ICU if the patient was intubated.

### Statistics

Sample size determination: The sample size was dictated by the number of participants in the trials from which these samples were provided.

Randomization and blinding: In MoDUS 142 participants were randomized 1:1 to receive 80 mgs of simvastatin or identical placebo enterally for up to 28 days.

Inclusion/exclusion criteria: Critically ill patients were eligible for MoDUS if they needed mechanically ventilation within 72 h of admission. The main exclusion criteria were current treatment with statin therapy, creatine kinase concentration more than 10 times upper limit of normal, alanine transaminase concentrations more than eight times upper limit of normal, uncomplicated elective surgery, expected discharge within 48 h, withdrawal of treatment within 48 h, estimated creatinine clearance of less than 15 msl/min and not receiving renal replacement therapy, no consent, allergy to statin drugs. The full exclusion list is in the supplementary appendix.

MoDUS plasma NfL levels were log-transformed in order to make them follow a Normal distribution for analyses, apart from comparison analyses between the control group and the MoDUS day one undertaken using raw data for Mann-Whitney test. The average plasma NfL levels were modelled using linear mixed model with fixed effects for the comparator groups, time and an interaction between time and comparator group. The comparison between the average plasma NfL levels at day 1 and day 3 were compared between comparator group using the marginal contrast in the model, the change between day 1 and day 3 were were compared between the groups using the interaction term in the model. The main comparisons are performed within the MoDUS group, and the comparison with the control group should be interpreted with caution, as these samples were collected in another setting and the measurements were performed in another round, albeit with the same method.

Correlation between plasma NfL levels and other continuous factors were based on a Pearson correlation coefficient, with assumption of linearity verified. Receiver operating characteristics (ROC) curve were estimated to measure the strength of using plasma NfL levels the predict outcomes. We included age, sex and chronic health for the 'baseline ROC' and then included NfL in the next one. We used the DeLong test to statistically compare the area under the curves for the two models.

A significance level of 0.05 was used to determine if the null hypothesis was rejected or not rejected.

In routine clinical practice many mechanically ventilated patients are deeply sedated such that delirium cannot be assessed on individual days. Deep sedation is an undesirable state, albeit sometimes required for clinical management. In addition many mechanically ventilated patients have at least one positive CAM-ICU delirium assessment on emerging from sedation.^22^ To make the results of this study more relevant to clinicial practice we included days in deep sedation as well as days in delirium. We also restricted the analysis to days in delirium in patients who never had days in deep sedation. See Tables E3 and E4.

Comparison 1: Patients who either did not have delirium days or deep sedation days and patients with one day of delirium or deep sedation compared with the rest of the study group.

Comparison 2: Patients who either did not have delirium days or deep sedation days and patients with one or two days in delirium and/or deep sedation compared with the rest of the group to explore the impact of an additional day with brain dysfunction on plasma NfL.

Comparison 3: Both patients with zero or one day in delirium and/or deep sedation with patients who were assessed with seven days of delirium or deep sedation as a definite indicator of prolonged brain dysfunction.

Comparison 4: Patients with zero or one day in delirium compared with patients who had (i) two days or more assessed with delirium and (ii) seven or more days with delirium. (Patients who were assessed as deeply sedated on any day were excluded from this analysis.)

Comparison 5: Patients with zero, one or two days in delirium with patients who had 7 or more days in delirium (patients assessed as deeply sedated on any day were excluded from this analysis).

For this study we used a baseline IQCODE cut-off score of 3.44 as an indicator of dementia.

### Role of funders

The sponsor of the study had no role in study design, data collection, data analysis, data interpretation, or writing of the report. VJP had access to and verified the pseudo-anonymized data, RDS and AC had access to and verified the anonymized data. The corresponding author (VJP) had final responsibility for the decision to submit for publication.

## Results

Between 1st February 2013 and 29th July 2016 142 patients were recruited to MoDUS. No patients in the cohort were admitted with a primary diagnosis of neurological disorder.

Trial participants demographic and baseline NfL levels (day 1) taken on admission to the study are in Table 1. 133 (93.7%) of the 142 participants identified themselves as white. There were a total of 142 study patients at baseline, 124 were still inpatients on the ICU on day 3 and 87 were inpatients on the ICU on day 7. The specimens collected on day 14 or day 28 were not included because of the small numbers at these time points.

**Table 1:** Baseline demographic, clinical characteristics and outcomes MoDUS patients.

	n = 142
Age (years)	62.0 (16.3)
Gender Male Female	82 (57.7%) 60 (42.3%)
Plasma neurofilament light protein (pgs/ml)	89.0 (101.6)
NfL level greater than normal limit according to age 18–50 yrs (normal < 10 pg/ml) 51–60 yrs (normal < 15 pg/ml) 61–69 yrs (normal < 20 pg/ml) >=70 yrs (normal < 35 pg/ml)	29/35 (82.9%) 20/23 (87%) 28/31 (90.3%) 45/53 (84.9%)
Sepsis on admission	41 (28.9%)
IQCODE Score IQCODE Score >3.44	3.3 (0.3) n=127 13.4% n=19
RASS Score Lowest Highest	-4(-4,-3) 1(-1,2)
CAM-ICU Status Positive Negative Unable to assess	112(78.9%) 4(2.8%) 26(18.3%)
APACHE II Score Correlation coefficient for association plasma NfL on day one with baseline APACHE II	17 (13-20) 0.5 p-value <0.001
PRE-DELIRIC: risk of delirium development (%)	70.9±25.7
Days in delirium to 14 days	5.6 (4.4)
Length of hospital stay until death or discharge from point of randomization (days)	20.4 (19.5)
All cause mortality 6 month post randomization	45/142 (31.7%)

Data are mean (SD), n (%), median (QR) NfL = neurofilament light protein, IQCODE = Informant Questionnaire on Cognitive Decline in the Elderly, RASS = Richmond Agitation and Sedation Scale, CAM-ICU = Confusion Assessment Method – Intensive Care Unit, APACHE = Acute Physiological And Chronic Health Evaluation, PRE-DELIRIC = PREdiction or DELIRium in ICu patients.

The mean day one NfL levels for all patients was elevated 89.0 pg/ml (SD 101.6) as compared with our control group of 68 preoperative patients undergoing major surgery mean 27.3 pg/ml (SD 25.6). There was no difference between the number of men and women between the control group patients and the MoDUS patients, 73/111 (65.8%) men vs. 82/142 (57.7%) p value = 0.19. Control group patients, however, were comparatively older than the MoDUS patients 72.1 years (SD 5.3) vs. 62.0 (SD 16.3) p value < 0.0001.The difference between the medians of MoDUS patients 55.8 pg/ml (IQR 25.8-114.8) and control group patients 20.5 pg/ml (IQR 16.0-29.0) was significant p < 0.001.

More than 82% of patients in all age groups had day 1 mean plasma NfL levels above normal reference limits for the laboratory i.e. 18 to 50 years less than 10 pg/ml, 51 to 60 years less than 15 pg/ml, 61 to 69 years less than 20 pg/ml, 70 years or older than 35 pg/ml.

### Days in delirium or deep sedation

Day 1 plasma NfL levels were higher in patients who then spent two or more days in delirium or deep sedation (n=123) compared with patients who spent only one or no days in delirium or deep sedation (n=18), mean 37.8 pg/ml (standard deviation 32.6) compared with 96.5 pg/ml (SD 106.1) p-value 0.002 (Table 2). Similarly, plasma NfL levels were increased on day three in patients who spent more than one day in delirium or deep sedation compared with patients who had no or one day of delirium or deep sedation, mean 37.0 pg/ml (SD 20.5) compared with 141.1 pg/ml (SD 206.4).

**Table 2:** Comparison of plasma NfL levels between patients according to days spent in delirium and deep sedation. (i) Zero or one days in delirium/deep sedation and two or more days in delirium/deep sedation (ii) Zero or one days in delirium/deep sedation and seven or more days in delirium/deep sedation (iii) Zero, one or two days in delirium/deep sedation and seven or more days in delirium/deep sedation

i.	0 or 1 days delirium/deep sedation		2 or more days delirium/deep sedation		p-value1
	N	Mean (SD)	N	Mean (SD)	
Concentration pg/ml day 1^1^	18	37.8 (32.6)	123	96.5 (106.1)	0.002^2^
Concentration pg/mlday 3^1^	11	37.0 (20.5)	113	141.1 (206.4)	<0.001^2^
Concentration ratio day 3 to 1	11	1.4 (0.5)	113	1.7 (1.2)	0.2^3^

ii.	0 or 1 days delirium or coma		7 or more days delirium or coma		p-value
	N	Mean (SD)	N	Mean (SD)	
Concentration pg/ml day 1^1^	18	37.8 (32.6)	62	94.17 (74.3)	<0.001^2^
Concentration pg/ml day 3^1^	11	37.0 (20.5)	62	141.7 (107.5)	<0.001^2^
Concentration ratio day 3 to 1	11	1.4 (0.5)	62	1.8 (1.3)	0.13^3^

iii.	0, 1 or 2 days delirium or coma		7 or more days delirium or coma		p-value
	N	Mean (SD)	N	Mean (SD)	
Concentration pg/ml day 1^1^	38	66.9 (77.9)	62	94.17 (74.3)	0.004^2^
Concentration pg/ml day 3^1^	23	59.0 (66.0)	62	141.68 (107.5)	<0.001^2^
Concentration ratio day 3 to 1	23	1.3 (0.4)	62	1.8 (1.3)	0.02^3^

^1^ based on a linear mixed model with log concentration as the outcome and fixed effect for delirium/deep sedation and day (1 or 3) and the interaction between delirium/deep sedation and day with a random effect for the participant including data from day 1 and day 3.

^2^p-value is the marginal contrast at day 1 and day 3 respectively.

^3^based on the interaction between delirium/deep sedation and day.

There were no differences between the change in concentration between day one to day three when comparing patients spending no days or one day in delirium or deep sedation with mean concentration ratio day three to day one of 1.4 (SD 0.5) with patients who had two days or more in delirium or deep sedation with mean concentration ratio day three to day one of 1.7 (SD 1.2) p-value 0.2.

In addition when patients who had one or no days in delirium or deep sedation (n=18) were compared with patients who had seven or more days in delirium or deep sedation (n=62) there was a highly significant difference at day three with mean plasma neurofilament light level of 37.8 pg/ml (SD 32.6) in patients with no or one day of delirium/deep sedation compared with 94.2 pg/ml (SD 74.3) in patients with seven or more days in delirium/deep sedation p<0.001.

Similarly when comparing patients with up to two days of delirium or deep sedation (n=38) with patients who had seven or more days in delirium or deep sedation (n=62) there was a difference at baseline that remained highly significant on day three. Day one plasma NfL levels were 66.9 pg/ml (SD 77.9) in patients with two days or less in delirium or deep sedation and 94.2 (SD 74.3) in patients with seven days or more in delirium or deep sedation.

There was also a difference between the two groups in the NfL concentration ratio between baseline and day three with mean ratio of 1.3 (SD 0.4) from day 1 to day 3 in patients with two days or less in delirium or deep sedation compared with 1.8 (SD 1.3) in patients with seven days or more in delirium or deep sedation.

These differences remain significant when the same comparisons are undertaken excluding those patients who were assessed as deeply sedated on any day. For zero or one day in delirium the mean concentration of plasma NfL was 37.8 pg.ml (standard deviation 32.6) as compared with 86.7 pg/ml (SD 97.1) in patients with two or more days in delirium p-value 0.006. For patients with zero, one or two days in delirium the mean plasma NfL was 54.2 pg/ml as compared with patients who had seven days or more delirium with mean level of 91.5 pg/ml (SD 76.0) p-value <0.001.

### Statin therapy

There was no difference in NfL levels in those patients who were given simvastatin as opposed to placebo either at day one or day three, day one mean plasma NfL levels in placebo group were 91.8 pg/ml (SD 109.2) and in the simvastatin treatment group 86.3 pg/ml p-value 0.95.

### Patient admission data

There was a moderate correlation between plasma levels of NfL and severity of illness as assessed using the APACHE II score on admission to ICU with a correlation coefficient of 0.47, p-value <0.001. When this association was adjusted for pre-existing dementia (IQCODE > 3.44), and diabetes the regression coefficient for baseline IQCODE was 0.29 (95% confidence limits 0.21, 0.79) and for diabetes was 0.05 (95% CI 0.6, 0.69) reducing the association but it remained significant (Table E1).

Significant differences in concentration at day one and three were observed between those with and without sepsis, those without sepsis having lower mean levels on both days with a greater difference between the groups on day three. On day one the mean plasma concentration of NfL was 76.5 pg/ml (SD 79.0) in patients without sepsis and 120.8 pg/ml (SD 140.2) in patients with sepsis, p-value 0.006, and for day 3 100.8 (100.2 without sepsis and 221.2 (342.1) with sepsis, p-value <0.001 (Table 3).

**Table 3:** Comparison of plasma NfL levels between patients according to days spent in delirium, excluding patients who were ever assessed with deep sedation.

i.	0 or 1 days delirium		2 or more days delirium		p-value
	N	Mean (SD)	N	Mean (SD)	
Concentration pg/ml day 1^1^	18	37.8 (32.6)	76	86.7 (97.1)	0.006^2^
Concentration pg/ml day 3^1^	11	37.0 (20.5)	70	117.5 (165.5)	0.003^2^
Concentration change (ratio) day 3 to 1	11	1.4 (0.5)	70	1.5 (0.6)	0.5^3^

ii.	0 or 1 days delirium		7 or more days delirium		p-value
	N	Mean (SD)	N	Mean (SD)	
Concentration pg/ml day 1^1^	18	37.8 (32.6)	33	91.5 (76.0)	<0.001^2^
Concentration pg/ml day 3^1^	11	37.0 (20.5)	33	129.7 (98.6)	<0.001^2^
Concentration change (ratio) day 3 to 1	11	1.4 (0.5)	33	1.6 (0.7)	0.207^3^

iii.	0, 1 or 2 days delirium		7 or more days delirium		p-value
	N	Mean (SD)	N	Mean (SD)	
Concentration pg/ml day 1^1^	35	54.2 (53.8)	33	91.5 (76.0)	0.005 2
Concentration pg/ml day 3^1^	23	9.0 (66.0)	33	129.7 (98.6)	<0.001^2^
Concentration change (ratio) day 3 to 1	23	1.3 (0.4)	33	1.6 (0.7)	0.071^-^

^1^ based on a linear mixed model with log concentration as the outcome and fixed effect for delirium/deep sedation and day (1 or 3) and the interaction between delirium/deep sedation and day with a random effect for the participant including data from day 1 and day 3.

^2^p-value is the marginal contrast at day 1 and day 3 respectively.

^3^based on the interaction between delirium/deep sedation and day.

**Table 4:** Sepsis Comparison of NfL plasma concentration levels between patients who did not have a diagnosis of sepsis on admission and those who did have a diagnosis of sepsis on admission.

	No Sepsis N		Sepsis N		p-value
Concentration day 1^1^	101	76.5 (79.0)	40	120.8 (140.2)	0.004^2^
Concentration day 3^1^	92	100.8 (100.2)	32	221.2 (342.1)	0.010^2^
Concentration ratio day 3 to day 1	92	1.8 (1.3)	32	1.5 (0.5)	0.704^3^

^1^ based on a linear mixed model with log concentration as the outcome and fixed effect for delirium/deep sedation and day (1 or 3) and the interaction between delirium/deep sedation and day with a random effect for the participant including data from day 1 and day 3.

^2^p-value is the marginal contrast at day 1 and day 3 respectively.

^3^based on the interaction between delirium/deep sedation and day

### Length of stay and mortality

A significant difference in concentration at day one was observed between those alive and discharged from hospital within 14 days 38.4 pg/ml (SD 40.3) compared to those who died within six months and/or had length of stay in hospital of 14 days or more, 107.7 pg/ml (110.8) p-value <0.001 (Table 5). A significant difference in mean NfL concentration at day one was observed between those who survived 65.8 pg/ml (SD 86.6) and those who died within six months 140.2 pg/ml (114.0).

**Table 5:** Adverse clinical outcomes (i) Comparison between NfL plasma concentration at day one and change day one to three in patients with hospital lengths of stay of over 14 days who survived 6 months with patients who had a hospital stay of less than 14 days and/or died within 6 months. (ii) Comparison between NfL plasma concentration at day one in patients who survived up to 6 months and those who died.

i.	Alive and LOS <14 day (n=38)	Dead or LOS >=14 days (n=104)	Effect size (95 % CI)	p-value
Day 1 Concentration pg/ml1	38.4 (40.3)	107.7 (110.8)	0.34 (0.2,0.5)	<0.001^2^
Concentration ratio day 3 to day 1	1.6 (0.8)	1.7 (1.2)	0.93 (0.8, 1.1)	0.445^3^

ii.	Alive (n=97)	Dead (n=45)		p-value
Day 1 Concentration pg/ml1	65.8 (86.6)	140.2 (114)	0.38 (0.3,0.5)	<0.001^2^
Concentration ratio day 3 to day 1	1.6 (1.1)	1.9 (1.3)	0.87 (0.7, 1.0)	0.145^3^

LOS = Length of hospital stay.

^1^ based on a linear mixed model with log concentration as the outcome and fixed effect for delirium/deep sedation and day (1 or 3) and the interaction between delirium/deep sedation and day with a random effect for the participant including data from day 1 and day 3.

^2^p-value is the marginal contrast at day 1 and day 3 respectively.

^3^based on the interaction between delirium/deep sedation and day.

**Figure 1:**
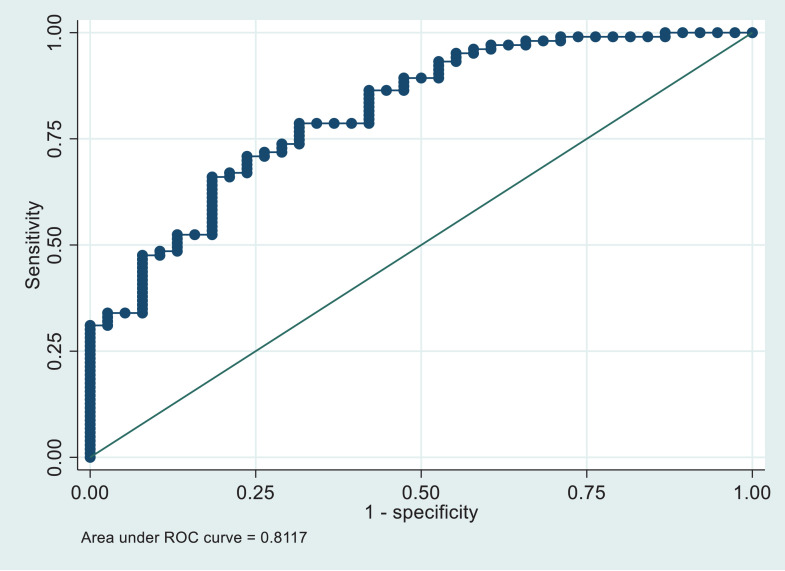
The ROC curve for LOS of 14 days or more or death on day one concentration and age, sex and chronic health is 0.81 (0.73,0.89). (i) ROC Curve: Day 1 plasma NfL concentration and patients with hospital length of stay over 14 days and/or died within 6 months. (ii). ROC curve: Day 1 plasma NfL concentration and patients who died within 6 months.

The area under the baseline ROC curve for predicting death within 6 months and/or length of stay in hospital of 14 days or more according to age, sex, and chronic health is 0.74 (95% confidence limits 0.65 to 0.84). By including plasma levels of NfL on day one this increases to 0.81 (0.73, 0.89) 0.81 (0.73, 0.89) p value 0.028 (Figure 1). There was no association between mortality and change in NfL concentrations between days one and three.

### Cognitive outcomes

There was no significant correlation between the BTACT composite score or any of the NfL measures after excluding patients with an IQCODE score of over 3.44 (cut off for dementia).

## Discussion

Increased plasma NfL is a highly sensitive but non-specific indicator of neuronal damage. In this study we have shown a positive association between plasma NfL levels on admission and delirium days plus deep sedation days in a general adult critical care population. Patients with higher plasma NfL levels on admission and day three had more days in delirium or deep sedation as well as poorer outcomes either as a hospital stay of 14 days or more and/or death by six months following recruitment. Baseline NfL levels were high as compared with a control group of hospital patients undergoing major elective surgery who did not develop post-operative delirium. Only 20 patients (14%) had plasma NfL levels in normal range, suggesting that most of the patients already had a degree of neuronal damage. The prevalence of abnormal plasma NfL level is likely due to the multiple drivers of encephalopathy with delirium seen in critically ill patients e.g. sepsis, metabolic, sedatives, hypoxia . We were unable to show an association between critical care plasma NfL levels and long-term cognitive outcomes possibly due to the small number of patients with six-month follow-up data, which was only forty-two.

Recent observational evidence of changes in total tau and phosphorylated tau correlating with changes in postoperative delirium severity support a link to dementia pathologies.^23^, ^24^ The same group also identified a link between amyloid deposition on positron emission tomography and delirium severity.^25^ These potential links show that delirium may be linked to dynamic changes in dementia pathologies that may explain the burden of cognitive decline incurred by patients with critical illness.

We showed a positive association between increase in plasma NfL levels with severity of illness and sepsis on admission. Both increased severity of illness and sepsis are recognised risk factors for increased duration of delirium.^26^, ^27^ In our study NfL levels already high at baseline rose further by day three. A small pilot study has showed a substantial increase in patients with sepsis as compared with patients without sepsis on day seven but no difference on day one.^28^ It may be their patients were admitted earlier to intensive care in their disease process than our study population and it is noteworthy the NfL levels continued to rise despite ongoing therapy for patients sepsis. The associations between NFL concentrations and sepsis, mortality and hospital length of stay are important but it is not possible to know if these factors contribute to any association with delirium when combined with deep sedation.

It is not known from this study if neuronal damage in our patients is a result of delirium pathophysiology, an encephalopathy directly related to the admission critical illness resulting in delirium symptoms, or a combination of both. In 2020 Casey and others showed delirium is associated with exaggerated increases in blood NfL levels in a post-operative patient population and investigated whether the change in levels attributed to delirium severity was independent of inflammation using IL-8 as a marker.^13^ They concluded that the presumed neurotoxicity indicated by high NfL levels may contribute to the pathogenesis of delirium, independent of changes in inflammation. More information is needed regarding the pathogenesis of neuronal damage resulting from neuroinflammation in the context of sepsis, dynamic changes in NfL levels and if changes are in part or independent of ongoing delirium.

While it is established that NfL increase is associated with postoperative delirium, the prevalence of delirium in critical care, the different precipitants and drivers of that delirium, and the associated adverse outcomes make this study an importance advance in delirium biomarker research.^12^, ^13^ A biomarker that links duration of delirium and outcomes would in part enable researchers to overcome the problem of heterogeneity inherent in most criticial care research. By being able to identify those patients likely to have short-lived delirium that spontaneously recovers, as well as those patients likely to have the worst outcomes researchers can target the participants and size of studies according to the aims of their research and their intervention.

Most studies designed to prevent or reduce delirium would not want to include patients who are unlikely to develop delirium or those who are likely to die within six months. There is, however, also important research needed in patients with short-lived delirium and those with expected poor outcomes. For example it has been demonstrated mechanically ventilated patients who have rapidly reversible delirium attributed to ongoing sedation have the same outcomes as patients who do not develop delirium.^29^ By comparing NfL levels in these patients during admission and long-term follow-up we would be able to investigate further if sedation is a risk factor for an increase in NfL with or without delirium. Conversely in patients with sepsis who have high NfL levels, which continue to rise, we need to promote innovative, anti-inflammatory targeted interventions as a matter of urgency.

It may be future research will find there is a level of blood NfL above which no intervention is likely to work to modify a patient's delirium, and in these patients rehabilitation or palliative care and research would be a priority. Overall changes in NfL levels taken at six months and 12 months would be interesting to explore if critical care survivors have potential for further functional and cognitve recovery once NfL either normalised or plateaued.

In this study plasma levels of NfL rose and remained high or continued to rise in patients who remained alive in the ICU for up to 28 days. A recent study in 142 COVID-19 critically ill patients compared with controls showed in some individuals, serum NFL rose over time, whereas for others NFL remained relatively consistent or less often fluctuated to varying degrees.^30^ Earlier studies in other patient populations have shown that NfL is slow to normalise. Following traumatic brain injury patients had NfL levels significantly increased from admission to neurointensive care unit to day 12, with the highest levels were measured at 12 days after injury.^31^ The levels normalized by 1-year follow-up. Similarly, a study in boxers suggests that it may take several weeks or months after a traumatic brain injury before NFL levels are normalized.^7^ Finally, a post-operative study showed that the delirium group was found to have higher NfL levels that continued to increase at one month after hospitalization, compared with the baseline level.^14^ We do not know how long plasma NfL levels remain high after the neuronal damage has stopped and recovery started. This is an important area for future research.

Authors of a review of neurofilaments as markers of neurodegeneration included a study in patients following cerebral infarcts and suggested the release of NfL after acute neuronal damage could be due to continuous breakdown of the blood–brain barrier, but persistent post-ischemic inflammatory and immunological processes could also explain lengthened NF-L release.^32^ In critically ill patients with sepsis these processes could be driven by ongoing inflammatory response. We need to know more about the dynamics of NfL release and how long after neuronal damage and release of into the blood that we can expect the levels to plateau and then fall. Only then can we determine the utility, if any, of serum NfL as an early response to intervention biomarker.

Relevant to critical care patients was a study in 1379 patients that used serum neurofilament light protein to further explore the mechanisms that underlie neuronal damage and cognitive dysfunction in atrial fibrillation.^33^ They showed that NfL was associated with worse cognitive performance, which was largely but not exclusively explained by age, co-morbidities, and vascular brain lesions. They also confirmed the strong independent association of NfL with age, which has been demonstrated across a wide variety of patient populations and healthy controls, probably reflecting neurodegenerative processes associated with normal ageing. Importantly, they found that diabetes mellitus was associated with a NfL increase by a similar magnitude as 10 years of age.

Our findings show that plasma NfL level taken in the first three days of intensive care admission has potential to identify patients likely to have seven days or more with brain dysfunction ie. in deep sedation or delirium in the first two weeks of admission, to have a hospital stay of a fortnight or more, and/or die within six months of ICU admission. This would help clinicians to advise patients, family members and primary care physicians of likely worse short term and long term outcomes, to be more realistic about individual patient's prognoses and enable better informed plans for patient's rehabilitation and follow up needs. Repeated measures in the hospital stay are unlikely to add any more useful information. Routine delirium screening will enable clinicians to determine the progress of acute cognitive recovery.

Measuring plasma NfL levels, however, at three- and or six-month follow-up with cognitive assessments would be extremely informative in determining the impact of interventional delirium research on long-term outcomes. It may also have a role in monitoring long-term clinical recovery over time for patients and family members.

The strengths of this study are the collection and storage of samples within a robust study and the use of a proven sensitive analysis of NfL levels completed in duplicate. We recognize that co-mingling delirium and deep sedation is problematic and a limitation. By incorporating days spent in deep sedation and delirium we were studying more than one state of consciousness. Even patients who did not record days in deep sedation would have experienced fluctuations in levels of arousal either due to an encephalopathy and/ or sedation. There are different states of consciousness critically ill patients experience over time resulting from being encephalopathic plus being in and out of deep sedation or delirium, none of which are desirable. This does not, however, mean that sedation-induced coma, deep sedation and delirium cannot be differentiated in scientific terms only that we are unable to confidently separate our patients into these states throughout their admission.

Our results do not prove that delirium causes neuronal damage resulting in a high NfL rather show that patients with high NfL on admission have more days in delirium and deep sedation, and worse clinical outcomes. Our hypothesis that NfL levels will change during ICU admission was not supported other than showing NfL levels remain high and increase over time in most patients. Our project cannot answer the question as to whether NfL concentrations at admission is simply a marker of vulnerability to delirium and worse clinical outcomes, whether the higher concentrations of NfL is solely related to the severity of the disease that got the patients admitted to the ICU in the first place, or whether delirium/deep sedation itself can cause permanent neuronal damage and worse clinical outcomes. This is a limitation with our study, and a limitation we share with most studies aiming to understand the relationship between acute illness and delirium.^34^

The weaknesses also include the relatively small number of samples taken after seven days, and the low numbers of follow up cognitive data. The majority of the participants identified as white and we were unable to make any meaningful comparisons based on ethnicity. The control sample data were historical, generated using the same methods, but a different kit lot and in another laboratory run by co-author Professor Zetterberg (University of Gothenburg). Therefore, comparisons between MoDUS results and the control group should be interpreted with caution. We did not adjust for pre-existing cardiac morbidities, diabetes mellitus or kidney dysfunction (all potential confounders of blood NfL concentration). While we cannot rule out the possibility that part of the NfL increase is the result of peripheral nerve injury it is unlikely to be the consequence of critical care polyneuropathy or myopathy given the timing of the samples.

While we cannot prove inter-rater reliability to evaluate delirium was consistent between the institutions providing the control and study data the same standard assessment tool, CAM-ICU, was used throughout. This is recommended for routine clinical use and research studies. Finally in this study relatively few patients had either no delirium or only one positive delirium assessment although overall days in deep sedation were low at mean number of 0.9, standard deviation 1.5 days.

This study shows that plasma neurofilament light protein correlates with delirium or deep sedation lasting more than one or two days, and unfavourable outcomes in a general adult critical care population. A NfL level taken within three days of admission will help identify patients with an increased risk of mortality or length of stay associated with increased rehabilitation needs following discharge. More research is needed into the dynamics of plasma NfL with regards to the trajectory of blood NfL levels from admission to long-term follow up. Larger studies are also needed with multivariable analyses to determine the extent to which the association of blood NfL levels and sepsis, mortality or length of stay are dependent on days in delirium with or without deep sedation. Future interventional delirium studies should consider either excluding or focusing on patients with sepsis, or at least plan an a priori analysis.

## Contributors

VJP, LOW, DMA and HZ conceived and designed the study. AH and RS advised regarding the sample analysis and collected the sample data. AC, VJP, LOW, DMA, RS and HZ contributed to data analysis and interpretation. VJP, LOW, AC, DMA, and HZ drafted the manuscript. All authors contributed to revision of the manuscript and approved the final version.

## Data sharing statement

Individual participant data that underlie the results reported in this article, after de-identification (text, tables, figures, and appendices) will be shared beginning three months and ending 36 months following article publication, to researchers who provide a methodologically sound proposal. Proposals should be directed to Fiona.smith8@nhs.net and to gain access, data requestors will need to sign a data access agreement.

## Declaration of interests

VJP, LOW, AH, AC and DFM declare no competing interests.

HZ has served at scientific advisory boards and/or as a consultant for Abbvie, Alector, Eisai, Denali, Roche, Wave, Samumed, Siemens Healthineers, Pinteon Therapeutics, Nervgen, AZTherapies, CogRx, and Red Abbey Labs, has given lectures in symposia sponsored by Cellectricon, Fujirebio, Alzecure and Biogen, and is a co-founder of Brain Biomarker Solutions in Gothenburg AB (BBS), which is a part of the GU Ventures Incubator Program.

## Appendix Supplementary materials

Image, application 1

Image, application 2
