# Supplementary material for: Plasma neurofilament light chain protein as a predictor of days in delirium and deep sedation, mortality and length of stay in critically ill patients
Source: eBioMedicine. 2022 May 6;80:104043. doi: 10.1016/j.ebiom.2022.104043 (PMC9092506; doi:10.1016/j.ebiom.2022.104043)
Supplement: Supplementary file 1 [file mmc1.docx]

**Supplementary Appendix**

**Plasma neurofilament light chain protein as a predictor of days in delirium and deep sedation, mortality and length of stay in critically ill patients**

Index

List of exclusion criteria for Evaluation of early administration of simvastatin in the prevention and treatment of delirium in critically ill patients undergoing mechanical ventilation (MoDUS): a randomised, double-blind, placebo-controlled trial

Table E1: Regression coefficients for association between APACHE and NfL concentration on day one adjusted for dementia and diabetes.

Table E2: Statin vs. placebo and NFL plasma concentration levels (pg/ml) by treatment group

Table E3: Number of study patients and days in deep sedation

Table E4: Number of study patients and days in delirium
