# Supplementary material for: Plasma neurofilament light chain protein as a predictor of days in delirium and deep sedation, mortality and length of stay in critically ill patients
Source: eBioMedicine. 2022 May 6;80:104043. doi: 10.1016/j.ebiom.2022.104043 (PMC9092506; doi:10.1016/j.ebiom.2022.104043)
Supplement: Supplementary file 2 [file mmc2.pdf]

Supplementary Appendix

Plasma neurofilament light chain protein as a predictor of days in delirium and deep sedation, mortality and length of stay in critically ill patients

Index

List of exclusion criteria for Evaluation of early administration of simvastatin in the prevention and treatment of delirium in critically ill patients undergoing mechanical ventilation (MoDUS): a randomised, double-blind, placebo-controlled trial

Table E1: Regression coefficients for association between APACHE and NfL concentration on day one adjusted for dementia and diabetes.

Table E2: Statin vs. placebo and NFL plasma concentration levels (pg/ml) by treatment group

Table E3: Number of study patients and days in deep sedation

Table E4: Number of study patients and days in delirium

Exclusion criteria for Evaluation of early administration of simvastatin in the prevention and treatment of delirium in critically ill patients undergoing mechanical ventilation (MoDUS): a randomised, double-blind, placebo-controlled trial

1. Age less than 18 years

2. Patient known to be pregnant or breastfeeding

3. Known allergy to statin drugs

4. CK > 10 times upper limit of normal range within 72 hours of randomisation.

5. ALT > 8 times the upper limit of normal range within 72 hours of randomisation.

6. Patients currently receiving ongoing and sustained treatment with any of the following; itraconazole, ketoconazole, HIV protease inhibitors, nefazodone, cyclosporine, amiodarone, verapamil, diltiazem, gemfibrozil or danazol

7. Uncomplicated elective surgery (planned admission, surgical procedure and recovery as predicted.)

8. Patient expected to be discharged within 48 hours of admission

9. Patients with severe renal impairment (estimated creatinine clearance less than 30ml/minute) not receiving renal replacement therapy

10. Severe liver disease (Childs-Pugh score >12)

11. Current or recent treatment (within 2 weeks) with statins as statins will be continued assuming there are no contraindications according to normal unit practice.

12. Physician decision that a statin is required for proven indication

13. Contraindication to enteral drug administration, e.g. patients with mechanical bowel obstruction. Patients with high gastric aspirates due to an ileus are not excluded.

14. Known participation in investigational medicinal product trials within 30 days

15. Consent declined

16. Treatment withdrawal likely within 48 hours

17. Non-English speaking patients or those who do not adequately understand verbal or written information

18. History of porphyria

Table E1: Regression coefficients for association between APACHE and NfL concentration on day one adjusted for dementia and diabetes.

	unadjusted				adjusted			
	Regression Coefficient (95% CI)[1]	p-value	Exponentiated Regression Coefficient (95% CI)	p-value	Regression Coefficient (95% CI)	p-value	Exponentiated Regression Coefficient (95% CI)	p-value
APACHE	0.09 (0.06,0.11)	<0.001	1.09 (1.06,1.12)	<0.001	0.09 (0.06,0.11)	<0.001	1.09 (1.06,1.12)	<0.001
Baseline IQCODE					0.29 (-0.21,0.79)	0.255	1.34 (0.81,2.21)	0.255
Diabetes (yes vs no)					0.05 (-0.60,0.69)	0.887	1.05 (0.55,1.99)	0.887
R-squared[2]	0.224	<0.001			0.231	<0.001		

[1]The regression was done on the log-transformed concentration but exponentiated coefficients are also provided.

[2]For adjusted model this is R-squared adjusted.

Table E2 Statin vs placebo and NfL plasma concentration levels (pg/ml) by treatment group

	Placebo	Simvastatin	p-value[1]
Day 1	91.80 (109.18)	86.30 (94.34)	0.95
Day 3	127.34 (177.25)	136.07 (219.29)	0.93

1.Two-sample t test based on log-transformed values.

Table E3: Number of study patients with days in deep sedation

Deep sedation	Frequency	Percentage	Cumulative
0	77	54.2	54.2
1	31	21.8	76.0
2	15	10.6	86.6
3	8	5.6	92.2
4	1	0.7	93.0
5	5	3.5	96.5
6	0	0	96.5
7 or more	5	3.5	100
Total	142	100	

Table E4: Number of study patients with days in delirium

Delirium	Frequency	Percentage	Cumulative
0	9	6.3	
1	22	15.5	21.8
2	19	13.4	35.2
3	14	9.9	45.1
4	11	7.8	52.9
5	5	3.5	56.4
6	6	4.2	60.6
7 or more	56	39.4	100
